# Parent Perceptions and Opinions of Universal Free School Meals in Arizona

**DOI:** 10.3390/nu16020213

**Published:** 2024-01-09

**Authors:** Sarah Martinelli, Emily M. Melnick, Francesco Acciai, Ashley St. Thomas, Punam Ohri-Vachaspati

**Affiliations:** 1 College of Health Solutions, Arizona State University, Phoenix, AZ 85004, USA; emily.melnick@asu.edu (E.M.M.); facciai@asu.edu (F.A.); punam.ohri-vachaspati@asu.edu (P.O.-V.); 2 Arizona Food Bank Network, Phoenix, AZ 85004, USA; ashley@azfoodbanks.org

**Keywords:** school meals, universal fee meals, healthy school meals for all, food policy

## Abstract

To support families during the COVID-19 pandemic, the USDA allowed all US schools to offer meals at no cost regardless of family income, a policy referred to as Universal Free Meals or Healthy School Meals for All (HSM4A). Despite the recognized benefits and popularity of HSM4A during the pandemic, the policy expired in June 2022. The goal of this study was to gather perceptions of parents in Arizona about school meals, the HSM4A program, and the discontinuation of HSM4A. In collaboration with a local anti-hunger group, using an online survey distributed in September and October 2022, we collected data from a diverse sample of over 2000 parents living in Arizona. Parents unequivocally supported HSM4A during the pandemic (97%) and expressed support for continuing to offer HSM4A (95%). High levels of support were seen across all groups in the study, including from individuals who identified as politically conservative. We also analyzed 750 responses to an open-ended question asking respondents to share their thoughts about offering meals to all Arizona students regardless of family income. The majority of emergent themes related to perceived benefits of HSM4A, including reducing financial burden and stress for families. Our findings will be useful for advocates and policy makers considering HSM4A legislation.

## 1. Introduction

Federal Child Nutrition programs such as the National School Lunch Program (NSLP) and School Breakfast Program (SBP) have long been a cornerstone of the federal food safety net in the United States. Since its inception in 1946, the NSLP was designed to “safeguard the health and well-being of the Nation’s children” [1]. The impact school meals continue to have on American families’ food security and well-being was brought into sharp focus in the early days of the COVID-19 pandemic when nationwide school closures limited students’ access to school meals, while, at the same time, the U.S. experienced unprecedented food supply disruption and job loss, as well as increased food insecurity, especially among underserved households and households with children [2,3]. To ensure school meals were able to continue supporting families during this time, the United States Department of Agriculture (USDA) issued a series of waivers that allowed school food providers to better reach students [4]. One such waiver removed the need for schools to collect income applications from families and allowed all students to receive free meals during pandemic-related school closures and throughout the 2021–2022 school year, when most schools resumed in-person learning. This policy is sometimes referred to as universal free meals or, as in the current paper, Healthy School Meals for All (HSM4A). 

Under current USDA guidelines, as well as those that were in place prior to the COVID-19 pandemic, schools participating in the NSLP and SBP are reimbursed for the meals they serve students through both (1) federal funds and (2) co-pays from students’ families. The reimbursement amounts are determined by a three-tiered eligibility system. In this system, the USDA’s reimbursement decreases as the student’s family income-based co-pay—the amount that schools charge families for meals—increases. Families submit annual applications to the school, reporting their family income and, based on these income applications, students fall into one of three categories: those who qualify for free meals, reduced-priced meals, or paid meals. Families that do not submit applications are automatically placed into the paid category. Schools are required to keep track of student meal eligibility category every time meals are served, collect family co-pays, and, when necessary, collect meal debt incurred by students who fail to make the co-pay at the time of procuring a school meal. This tiered system can create an environment of stigma for both parents and students and tends to increase application barriers for families that could benefit from the program [5,6]. 

Following the de-implementation of nationwide HSM4A waivers, some states took action to ensure continued free meal access for all students. California and Maine acted quickly to pass state-level legislation to permanently implement HSM4A, while Vermont, Massachusetts, and Connecticut enacted temporary laws to maintain HSM4A school meal access for the 2022–2023 school year [7]. Some states instituted measures that applied to specific student groups. For example, Arizona implemented a policy that only covered the co-pay for families who qualified for reduced-price meals, while it did not cover the co-pay for families in the paid meals category [8]. There is continued momentum in several other states to pass HSM4A legislation; however, most states have returned to pre-pandemic operations and the three-tiered system [7]. 

Over three out of four (78%) parents in Arizona reported that the meals were helpful for their families when they were asked about free school meals distributed during the summer of 2020 [9]. Similarly, a sample of parents in California responding to a survey in November 2020 reported that free school meals distributed during the pandemic helped their families financially by allowing them to save money on groceries and that the meals contributed to filling hunger gaps [10]. In both studies, parents also reported some concerns with the quality of food served [9,10]. Following the de-implementation of HSM4A programs across the country, it is important to understand the perspectives of students’ families in states that did not continue to offer free meals to all students. Gauging parents’ support or lack of support for HSM4A may help decision-makers as they assess the need for expanding school meals in the state of Arizona. In this cross-sectional study conducted immediately following the reinstatement of the three-tiered payment system, when schools were once again required to collect income applications, we examined the opinions and perspectives of parents of school-aged children in Arizona surrounding HSM4A legislation both during and after the pandemic. 

## 2. Materials and Methods

### 2.1. Survey Instrument

A 29-item survey instrument was developed to collect the opinions and perspectives of parents (and in some cases caregivers, such as grandparents) of students attending Arizona primary and secondary public schools. The survey questions were adapted from previously developed survey tools that were reviewed by experts in nutrition, public health, and by local advocates working on school food environment issues. These questions were used to collect similar data in other states [11,12,13,14,15]. Respondents were first asked multiple close-ended questions about their views on school meals, their level of agreement with statements on a variety of topics related to school meals (e.g., school meal healthfulness and whether school meals reduce family stresses), their level of support for the federal policy that allowed schools nationwide to offer free meals to students during the COVID-19 pandemic, and whether they would support a potential future extension of such policy in the state of Arizona. The survey also asked respondents to report demographic information (e.g., race and ethnicity, annual family income, and education level) and their political leanings. One open-ended question was included at the end of the survey, “Please provide any additional thoughts, opinions, or experiences you may have about offering school meals at no charge to all Arizona students regardless of family income”. The full survey instrument is provided in Supplementary File S1. 

### 2.2. Data Collection

**Figure 1:**
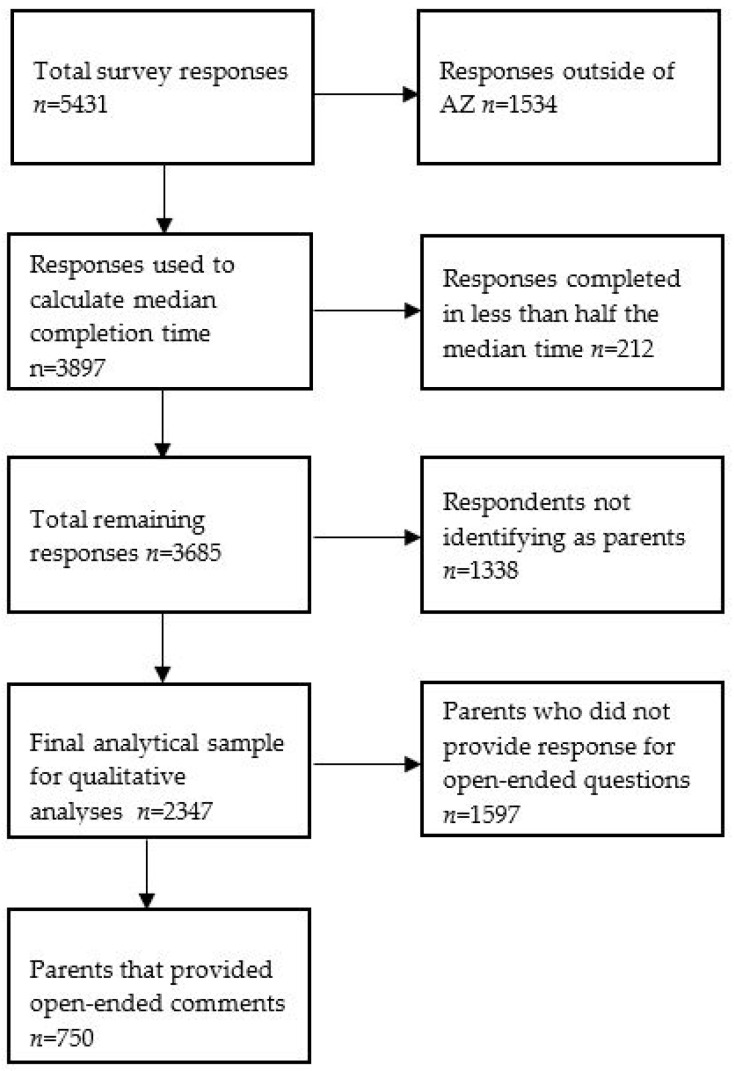
Description of study sample.

The survey was open for completion in September and October of 2022, which was two months after the de-implementation of HSM4A nationally and in Arizona and 2 months before state policy that removed the co-pay for families eligible for reduced-price meals was put into place. The survey was administered through the Qualtrics online platform (Qualtrics International Inc., Seattle, WA). The survey was distributed primarily through 6 school districts in Arizona, with distribution methods varying slightly based on district policies and procedures. The most common distribution methods included sending emails to school community members and posting on school-managed social media platforms. In addition, the Arizona School Nutrition Association shared the survey link to its members via email and study team members shared the survey on social media platforms (e.g., Twitter (now X), Instagram). Respondents consented to participation before answering survey questions and were given the option to provide their email address at the end of the survey to be entered to win one of five USD 100 gift cards, following the completion of the survey. On average, the survey took 13 min to complete. All procedures were approved by the Arizona State University Institutional Review Board. In total, 5431 responses were collected. Prior to analyses, the study team completed steps to ensure the quality and integrity of the data. First, using geographic information systems (GIS) software, we identified and removed responses from locations that were outside of Arizona, based on the latitude and longitude provided by Qualtrics. The purpose of this was to ensure that all responses were valid responses from Arizona residents. In this step, a total of 1534 responses from locations outside of Arizona were removed from the sample. Surveys completed in less than half the median time were also removed from the sample (n = 212). Of the remaining 3685 respondents, 2347 self-identified as parents and were included in descriptive analyses. Of those, 750 provided a comment in the open-ended question and were included in qualitative analyses (Figure 1).

### 2.3. Data Analysis

Descriptive statistics were calculated for close-ended survey questions using Stata software version 16 (StataCorp LLC, College Station, TX, USA). Responses to the open-ended survey question asking respondents to provide additional thoughts, opinions, or experiences surrounding offering free school meals to all students were reviewed and analyzed to develop thematic codes. As a first step, two researchers independently read a selection of 5% of the 750 responses. They then collaboratively developed a codebook with names and definitions for agreed upon codes. Next, both researchers independently coded a second selection of 75 open-ended responses (approximately 10% of total responses). Intercoder reliability was high at this stage (greater than 75% percent agreement on all applied codes). The two researchers subsequently met and discussed any discrepancies in assigned codes to reach consensus. The remaining 85% of the responses were reviewed and coded by a single researcher. After coding was completed, the researchers identified and named emergent themes by analyzing coded data, in line with systematic thematic analysis frameworks [16].

## 3. Results

### 3.1. Sample Characteristics

About half of the full analytical sample (i.e., 2347 parents) self-identified as Hispanic (45.9%), 3.2% as non-Hispanic Black, 3.9% as American Indian/Alaskan Native, and 40.5% as Non-Hispanic White (Table 1). Nearly 40% of the sample had a 4-year college degree or higher, while 40.4% of respondents completed at least some college or had an Associate’s degree. A little over one-third of respondents reported an annual income of less than USD 34,999 (36.4%), 27.7% reported an annual income between USD 35,000 and USD 64,999, 16.6% reported an income between USD 65,000 and USD 99,999, and 19.4% reported an income of USD 100,000 or greater. Respondents came from across the political spectrum with roughly one-third (35.0%) identifying as liberal, 30.7% being middle of the road, and 18.6% identifying as conservative.

### 3.2. Closed-Ended Survey Results

**Figure 2:**
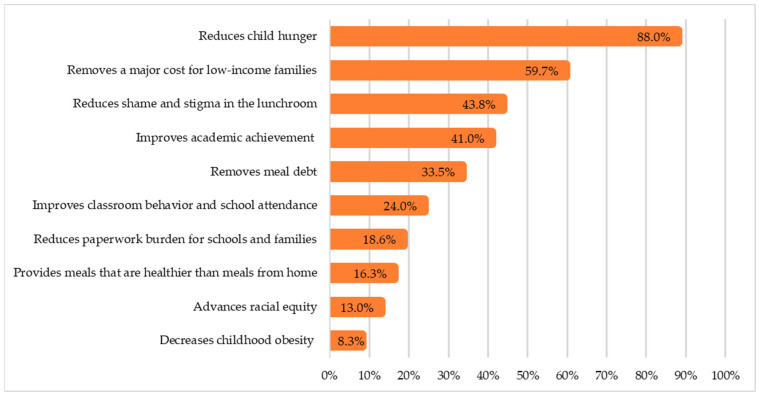
The most important benefits of making school meals available at no change regardless of family income, selected by parents (*n* = 2347).

Nearly all respondents (97.4%) indicated support for the distribution of free meals to all students during the COVID-19 pandemic. Similarly, 95.4% of respondents indicated support for continuing to offer free meals in Arizona for all students in the future. In response to a question asking respondents what policy actions to improve access to school meals they were most likely to endorse, 81.5% of parents identified HSM4A as the best approach for providing school meals in the future, while the second most selected response (14.2%) was to modify the eligibility criteria so more children would qualify for free or reduced-price meals. When parents were asked about the benefits of making school meals available to all students at no cost, parents selected reduced hunger (88.0%), lower costs for low-income families (59.7%), and reduced stigma associated with participating in school meals (43.8%) as their top three choices (Figure 2). Finally, when asked about why their children may not participate in school meals daily, the top reported reason was that children report not liking the taste of certain meals (46.4%) followed by menu fatigue (27.8%) and concerns about the healthiness of meals served (21.1%). Responses to additional questions asked in the survey are summarized in Supplementary File S2. 

### 3.3. Open-Ended Survey Results

Thematic analysis of open-ended survey responses yielded several overarching themes that summarize Arizona parents’ perceptions and opinions of HSM4A. These themes, along with associated relevant quotes, are described in the text below and are presented in two tables—one reporting reasons to support HSM4A (Table 2) and the other reporting concerns about HSM4A (Table 3).

#### 3.3.1. Reasons to Support HSM4A

**Reduces financial hardship for families.** A theme emerging from parents’ responses was that financial struggles persist for families even after the end of the COVID-19 waivers from the USDA providing meals to all students at no cost. They credited the HSM4A program that was in place during the pandemic with helping reduce their household financial burden. For example, one parent shared, “*School meals help my family because I can’t afford really anything barely my bills* [sic]*. I don’t want my child or anyone else’s* [sic] *to be hungry*”. Financial difficulties also exist among families whose children do not qualify for free or reduced-price meals. One caregiver shared, “*Although my income is above the limit it is still a struggle to send my granddaughter to school with a lunch every day. Medical bills, higher cost of everything including food, and family emergencies have all zapped my income*”. 

**Reduces family stress.** Parents also shared that HSM4A reduced their family’s stress load by removing the daily stressors of packing lunches and/or loading money onto their child’s lunch account. For example, one parent highlighted the logistical barriers to ensuring their students had meals on top of financial barriers, writing, “*Having school lunches as an option removed a daily stressor in our household. No more having to remind multiple people multiple times to 1) pack a lunch and/or 2) take it with them to school. It also saves my family at least $260 per month. That’s the cost of our electric bill during the summer or our gas bill in the winter. I’d be happy to pay more in taxes to have someone take this issue off my plate*”. Another parent shared, “*I used to pay for 2 kids to buy lunch at school and it was expensive and a hassle. We are a two income household and never qualified for free lunch before COVID. The free meals made a huge difference for our home and gave me one less thing to stress about…*”. 

**Reduces application barriers**. Another theme that emerged based on responses from parents was that the elimination of the application process for free or reduced-price meals as a part of the HSM4A policy removed a barrier for families to access meals. One parent shared, “*The current application process for free or reduced lunch is inaccessible for people with disabilities, English not primary language*, etc. *This deters people from filling out English applications and going through processes*”. Many school districts have transitioned to online applications; however, this may present barriers to parents with lack of access to the internet or lack of technological fluency. For example, one respondent shared, “*Online application for school lunch program is difficult for grandparents and those who do not have no access to internet*”. In addition to reporting possible access challenges, parents expressed that HSM4A policies removed several barriers to application, including parents’ lack of engagement in their child’s education, discomfort with sharing detailed financial information with the school, and feelings of shame when admitting to the school that they need help feeding their family by completing application forms.

**Reduces stigma and provides a sense of community.** The potential shame parents might feel submitting a meal application, as well as students and families’ sense of stigma, may present a barrier to participation in school meals. Even students who qualify for free or reduced-price meals and who might need the support still choose to not participate. Under the current system, where most students who eat school meals are those who qualify for free or reduce-price meals [17], eating at school may present a marker of economic status. Parents surveyed felt that the implementation of HSM4A eliminated this sense of shame and stigma. One parent shared, “*My children are happy not to be single*[d] *out in front of other students*”. Moreover, parent comments about stigma included statements about an increased sense of community in the school when all students were able to eat meals for free. For example, one parent shared, “*If all students, regardless of family income, eat school meals, the stigma of school meals will disappear. Income-based classification of students can lead to new shame and low self-esteem. When all students have school meals, it enhances unity and strengthens social cohesion*”. Parents expressed their belief that removing this stigma would reduce students’ level of worrying about something (i.e., family income) out of their control. 

**Food at school is a basic right.** Another emergent theme was that parents endorsed the idea that access to food is a right for children, regardless of their parents’ income. One parent wrote, “*Every kid deserves to eat. No matter how much money the parents make…But with free food every kid no matter what has the opportunity to eat and do the best they can in class*”. Parents expressed a belief that schools have a special responsibility to ensure students basic needs are being met during the school day and that these needs include access to school meals, “*children deserve to be cared for (fed) while in the hands of their school, food is a basic need that should be given no questions asked*”. Another common theme was the idea that other groups in the care of the state or the federal government are guaranteed meals and children should also have this same support “*…Why should prisoners get free meals when our children don’t?*” 

**Improves child food security and academic achievement.** Another common theme was the belief that a critical benefit to HSM4A is improved student food security. For example, one parent wrote, “*There is so much food insecurity in Arizona and in the US. We proved during COVID that we can provide free lunches to all kids, and we should continue to do so*”. Further, parents related food insecurity to student worries and academic performance; one parent wrote, “*Students shouldn’t have to worry about being hungry at school. They will focus more and be able to learn better if they aren’t hungry*”. 

**Improves lunch food variety.** Finally, parents reported that HSM4A policies ensured not only improved access to food but also improved lunch variety for their children. Parents reported struggling to provide healthy foods for their children in packed lunches due to limitations on what can be stored in a lunch box when there is no refrigeration available or the ability to warm meals in the cafeteria. For example, one parent shared, “*My family doesn’t qualify [for free/reduced price lunch], and it would be helpful for [my son] to have health[y], hot meal choices instead of carrying his lunch all day. I have to pack room temperature non- perishables because his backpack is out in the hot weather a lot of the day before lunch*”.

#### 3.3.2. Concerns about HSM4A

**Program Implementation Challenges.** The key concerns about HSM4A expressed by parents were related to program implementation challenges, including the possibility of reduced food quality, longer lunch lines, not enough time allocated for lunch periods, and increased food and materials waste. As an example, one parent shared concerns about the food quality, “*If there will be money invested in feeding children for free at school, that food should be high quality food that children will want to eat. It would be wasteful to invest a lot of money in food that will ultimately go in the trash because it is low quality*”. Some parents also reported concerns about foods being overly processed and not always meeting the cultural preferences of their children. In addition to concerns about food quality, there were also concerns about long lines in the cafeteria and not enough time for students to eat their meals. One parent wrote, “*Lunch lines are often too long for my child to actually eat lunch most days*”. 

In addition, parents expressed concerns about both food and packaging waste in school meals because of HSM4A policies. Some parents offered ideas for reducing waste, for example, one parent wrote, “*I would like to see less waste. For example, if students don’t eat things from their lunches or breakfast, there needs to be a share table. And if there are still things on the share table at the end of the day that are not allowed to be re-stocked, they should go to school food pantries, be allowed to be picked up by families after school, go to shelters or some other way to get to food insecure people*”. Notably, even among parents who shared concerns about food quality, lines, and waste specifically related to the implementation of HSM4A, there was support for the program while also acknowledging room for improvement, for example, “*Wasted food and planning is a concern, but the idea of every child having two guaranteed meals per day is a wonderful use of taxpayer dollars. I think feeding them all will reduce stigma, simplify logistics and is the best way to ensure that those who need the food, get it*”. 

**Unfair costs to taxpayers.** Finally, parents reported concerns about the potential costs to taxpayers due to HSM4A policies. As an example of these sentiments, one parent wrote, “*My taxes help contribute to these programs. I shouldn’t be assisting in feeding other families*”. This sentiment was sometimes paired with the idea that the current income limits should be raised to allow more families to qualify for the meals, while maintaining a paid meal category for families with higher incomes, instead of providing free meals for all families; for example a parent wrote, “*I fully support no charge for school meals but not for everyone. I’d support raising the income level for families to qualify without a doubt. I have no interest in subsidizing lunches for those families that can afford to pay*”.

## 4. Discussion

In this study, a diverse sample of parents from six school districts in Arizona provided their perspectives about the benefits and concerns relating to implementing HSM4A during the COVID-19 pandemic and potentially in the future in Arizona, through both closed and open-ended questions. In closed-ended responses, an overwhelming majority of parents surveyed expressed strong support for continuing to offer free school meals to all children. These results were supported by the wide variety of reasons parents provided for supporting school meals in open-ended responses, including benefits to their own families, such as reduced daily stresses and benefits to the school community, such as a reduced sense of stigma surrounding participating in school meals. Key concerns were related to program implementation and taxpayer costs. 

The results from descriptive analysis of closed-ended questions, which showed that the vast majority of parents supported HSM4A legislation, align with findings from a survey conducted by the Urban Institute in December of 2021 and with a study examining opinions of parents of school aged children in Massechusetts. In the Urban Institute’s nationally representative sample of adults ages 18–64, 70% expressed support for permanent free school meals for all [18], whereas in Massachusetts, 84% of parents of school aged children reported support for HSM4A in their state [19]. While the support for school meals reported by the Urban Institute is high, it is lower than the results presented in the current study and those reported in Massachusetts. This difference might be due to the composition of the samples used in the two state-level studies, which only included parents of children currently attending school. Even some parents in our sample who did not endorse HSM4A as the best choice for schools going forward still indicated that they supported lowering eligibility criteria, allowing more families to qualify for free- and reduced-priced meals. 

It was clear from both open-ended and close-ended responses that as pandemic-era programs came to an end, some Arizona families were still feeling the financial burdens associated with continued supply-chain disruptions and inflation. An emergent qualitative theme echoed by parents, including those who did not qualify for free or reduced-price meals under current guidelines, was that HSM4A offered during the pandemic reduced household financial hardships. Correspondingly, 60% of respondents identified the removal of a major cost for low-income families as one of the most important benefits of making school meals available at no charge to all students. This is perhaps unsurprising given that the average wage in Arizona is 3% lower than the national average [20], while the cost of living is approximately 4% higher [21]. Further, the living wage calculator developed by Glasmeier and colleagues [22] indicates that the household income needed to support a four-person household (two adults and two children) in Arizona is upwards of USD 80,000, while the USDA’s household income guideline to qualify for free meals as a family of four is USD 39,000 [23].

Beyond the reduction of financial burden, parents found HSM4A to be helpful in reducing the level of daily stress surrounding planning, shopping for, and packing meals, a theme that was also seen in the close-ended questions. Specifically, parents mentioned challenges related to sending their children to school with high-quality, healthy foods, as most schools do not have temperature-controlled storage space for homemade lunches. Parents also reported less stress associated with dealing with the online payment systems, remembering to add money to their student’s account, and the fear of their children being refused a meal if that account ran low. Other quotes described reduced stress felt by their children as a result of HSM4A. Parents felt that children were less stressed when they did not worry about potential judgement or shame associated with eating school meals or being given alternative meals if their lunch account was not up to date. The focus parents put on stress reduction as a benefit of HSM4A is an interesting and new finding not yet discussed in the literature. Prior literature has largely focused on the role of school meals in improving dietary quality, food security, and/or student achievement [24,25]. Finally, parents also felt that everyone being treated equally in the cafeteria allowed for a more supportive environment and a sense of community within the school as a whole, consistent with past literature identifying the stigma of receiving free school meals as a barrier to participation in the program [5,26,27], as well as evidence supporting improved perceptions of their school environment when HSM4A programs are in place [28]. 

While many emergent themes from qualitative analyses highlighted perceived benefits of HSM4A in the viewpoint of parents, other themes highlighted concerns related to program implementation; specifically, concerns about increased food waste, food packaging, and longer lunch lines. It is possible that some food quality and packaging concerns identified by parents could be related to supply chain disruptions experienced since the start of the COVID-19 pandemic [14,29]. Concerns about food quality are in line with other studies, as many parents hold somewhat negative perceptions of the general food quality and variety of school meals, with many expressing a desire for more freshly cooked meals [30,31]. Findings suggest that improved funding for school nutrition programs to improve food quality, for example, by providing additional resources to support the purchase of kitchen equipment to batch-prepare fresh meals, may be warranted. Similarly, concerns over long lines and insufficient time for lunch were expressed even before the implementation of HSM4A legislation [32] and have been shown to impact students’ overall nutrient consumption during lunch [33]. School schedules and allotted times for lunch, which are set by the school administration, may need to be adjusted in the future to allow for sufficient service and eating times. It is suggested that a minimum of 20 min of seated time after a student obtains their food should be provided, which would equate to an approximate lunch period of 30 total minutes [34]. Less than half the schools in the US are meeting that expectation [35]. A number of parents provided creative potential solutions for food waste that they feared may increase with all students receiving free meals in open-ended responses. Suggestions included increased use of share tables (where students can take food items that other students do not want), taking leftover food to local homeless shelters, or packing up leftovers for families at risk of experiencing food insecurity to take home. 

### Limitations

This study summarizes the views on HSM4A from a diverse sample of Arizona parents from various educational, political, and income backgrounds. The response rate from parents in rural areas was notably lower than in urban areas within southwest Arizona, an area that was over-represented in the survey sample. Therefore, it is possible that the perspectives of parents in less-represented counties and areas may differ from those presented in this manuscript. Additionally, these findings may not be generalizable to other settings in the US or to the overall US population. Lastly, as with all survey research, individuals who chose to complete the survey may hold different opinions than those who did not participated in the survey. 

## 5. Conclusions

Parents in Arizona from a diverse set of backgrounds strongly supported increased access to school meals, with a strong preference for HSM4A legislation. Parents felt that universal free school meals offered during the 2020–2021 and 2021–2022 school years provided financial benefits to families, reduced stress for parents and students, and helped foster stronger school communities. Identified reasons to not support HSM4A legislation included increased waste and burden on taxpayers. Findings may be useful for policy makers, community stakeholders, advocates, and school districts considering HSM4A legislation.

## Figures and Tables

**Table 1:** AZ School Community Perspectives Survey Sample Demographics for Respondents Who Self-selected as Parents (*n* = 2347) and Those Who Provided Open-ended Responses (*n* = 750).

		All Parents		Parents with Open-Ended Responses	
		*n*	(%)	*n*	(%)
*n*		2347	(100.0)	750	(100.0)
Race/Ethnicity					
	Hispanic	1061	(45.9)	326	(43.9)
	Non-Hispanic White	936	(40.5)	299	(40.3)
	Non-Hispanic Black	73	(3.2)	23	(3.1)
	Non-Hispanic American Indian/ Alaska Native	91	(3.9)	34	(4.6)
	Non-Hispanic Other/Multiple	151	(6.5)	60	(8.1)
Education					
	High school diploma or less	509	(21.8)	142	(19.0)
	Some college/Associate’s degree	944	(40.4)	273	(36.5)
	4-year college degree	434	(18.6)	169	(22.6)
	Professional/PhD	451	(19.3)	165	(22.0)
Income					
	<$35,000	833	(36.4)	259	(35.3)
	$35,000–$64,999	634	(27.7)	189	(25.8)
	$65,000–$99,999	379	(16.6)	126	(17.2)
	≥$100,000	443	(19.4)	159	(21.7)
Political Affiliation					
	Conservative	431	(18.6)	137	(18.5)
	Middle of the road	714	(30.7)	219	(29.5)
	Liberal	814	(35.0)	278	(37.5)
	Not sure	365	(15.7)	108	(14.6)

**Table 2:** Emergent themes related to reasons to support HSM4A reported by parents of students attending public schools in Arizona (*n* = 750).

Theme	Quotes Representative of Key Themes
Reduces financial hardship for families	“I am not considered low income. I don’t qualify for any food stamp benefits. Our child support isn’t consistent, and my bills are way more than I make. Without free lunch I struggle and [my] kids are in debt with the price of daily lunch. Life is already difficult, and they shouldn’t have to worry about whether or not they can afford a school meal. Free school lunch would help out a lot of families like our[s] and relieve some of the stress and/or embarrassment our children experience when they don’t have money for a decent meal”. “$19 is how much [our income is over the limit to qualify] for free or [reduced-price] lunches. $19 dollars doesn’t even cover a week or lunches for my child. I barely make more than the allowed amount, but my cost for food for lunches is triple that. BRING BACK FREE LUNCHES FOR ALL”. “I make 70 k a year, I’m a single father, and my bills are so high I barely get by and I was denied school meals for my son this year because I make above the limit. So far this school year there are days where I didn’t have the money needed for my son’s lunch… If it affects me at my income level, imagine how many families are struggling to provide lunch money for their children. It’s so sad. No kid should go hungry. [For] a lot of kids in poverty, this is the only meal they might have all day”.
Reduces family stress	“As a mother whose income is low but not low enough to qualify, this program helps me tremendously. I go to school full time and work part-time, so I do not have the time to shop and prepare meals to send for lunch. I don’t qualify for food stamps but barely make ends meet. This gives me the security to know that my children are well fed in school”. “Paperwork required time to complete to qualify [and] adding funds for reduced meals was sometimes tedious and embarrassing; when we forgot and our child was denied a meal. Packing a lunch requires time and stress if we didn’t have items to pack. Now all we worry about is their education and focus on helping them with homework rather than trying to prep lunches or add lunch items to the grocery list. It’s more than time and money we save, it’s a huge relief to be able to send them knowing they will be fed healthy meals and not a lunchbox of poptarts and chips because that’s all we had or had time to grab”.
Reduces application barriers	“Having done the application for free lunch program, I know it is time consuming, annoying to have to do every year, [and] requires computer literacy, parental attention, involvement and capacity to apply. I would rather see some kids get a free lunch maybe don’t “deserve” than see any children lacking food for any of the barriers that exist”. “I believe there are a lot of children who would benefit from free meals but their families have a difficult time completing the process of filing for them due to language barriers, confusion filling forms out, home life where care givers are absent/abusive [and not] looking out for their [children’s] needs and taking steps to apply for free lunch, fear from immigration status and getting government involved, [and] shame around not being able to afford [meals] but not wanting school to know. There are so many reasons it’s difficult for families to even apply for help”.
Reduces stigma and provides a sense of community	“I think it helps children socialize and relate to each other by sharing in the same foods, which helps solidify unity amongst their peers”. “It helps children to have something else that all students have. Just like uniforms. It takes away status, income restrictions, and the stigma. Let them be kids and not worry about extra things they cannot change, like their families income/means”. “We don’t need or rely on school meals or snacks, but my son [is] on his high school’s basketball (varsity) team and frequently went through the lunch line last year to get extra meals for his teammates… There is quite a bit of stigma attached to going through those lines, and some of the boys wouldn’t do it. But my son knows [that] everyone else knows he doesn’t need the food, so…it’s not socially stigmatizing for him to be in the food line”.
Food at school is a basic right	“I believe it is more important to make sure our children are fed, regardless of income, than to argue about who should receive the meals. There are no negative consequences to feeding our children but there are innumerable consequences by allowing them to go hungry”. “No child should be hungry, particularly while at school, regardless of income”. “School districts do not charge students to take the bus to school… I see transportation and food as basic necessities. There are many middle-class 2-parent working families that are barely making ends meet right now that need help. Food prices are the highest they have been in 40 years. So much good was done during covid [sic] providing free meals to students. It is an optional/voluntary program. Parents can still make their kids meals if they want”.
Improves child hunger and academic achievement	“As a single mother, free/reduced meals have saved my children from food scarcity issues”. “I am a current public school teacher in a different district than my son attends. I know that food insecurity is a big deal for several of my students, [and] offering them a meal that they do not have to worry about paying for gives many of them peace. I have also seen the academic difference in a student that eats regular meals and a student that has food insecurities. It makes a difference in the classroom for all students!” “It is so important that we don’t let our kids go hungry. It is not their fault [that] their parents can’t or don’t have the means to pack them a lunch or buy them a lunch. A kid can’t concentrate on learning while their stomachs are hungry for food. In giving free lunch to all children, no child will go hungry and they will want to be in school and want to learn and apply themselves more”.
Improves lunch food variety	“I like the variety that the school lunch menu provides for both of my children. They enjoy this variety as well. The food is the appropriate temperature, unlike a lunch that has been sitting in a backpack. It is difficult to pack a lunch that consists of the same quantity as a school lunch. Currently, I have a high school student and a middle schooler”. “My family doesn’t qualify [for free/reduced price lunch], and it would be helpful for [my son] to have health[y], hot meal choices instead of carrying his lunch all day. I have to pack room temperature non-perishables because his backpack is out in the hot weather a lot of the day before lunch. It would be helpful to know how food was safely prepared and fresh”.

**Table 3:** Emergent themes related to concerns about HSM4A reported by parents of students attending public schools in Arizona (*n* = 750).

Theme	Quotes Representative of Key Themes
Program Implementation Challenges	
Subtheme: Food quality	“I think schools should focus more on the quality of food being offered to students. There is no point in offering free meals to all students if none of the students like the food. I think the quality of school lunch has gone down since the pandemic”. “The quality of the food provided when the [p]andemic started went down dramatically. My children won’t eat the foods because it tastes terrible. Quality needs to be addressed”.
Subtheme: Lunch lines and time allocated for lunch	“When lunch was free to everyone, the amount of time my child had to wait in line was long. So, the amount of time to eat it was too short and she never was able to finish it (thus wasting the meal and also staying hungry). I decided to start making her meals again even though the lunches were free, so my child had more time to eat and not spend a portion of it waiting in line”. “Lunch times need to be lengthened to accommodate more kids getting lunch. Would rather lunches be something kids will eat rather than being super healthy. No kid should be hungry”.
Subtheme: Food waste	“Universal meals contributes to a lot of wasted food. Some kids will only eat 1 or 2 items of everything that is given to them or get it because they can and throw the entire thing in the trash”. “There is so much waste when you are anticipating 1000 students for lunch from one high school when the lines are so long you barely get time to eat it so most of the food goes to waste. Students bring their lunches to avoid that issue. Schools making 1000 lunches are not going to put out tasty, delicious healthy food”.
Unfair costs to taxpayers	“I fully support no charge for school meals, but not for everyone. I’d support raising the income level for families to qualify without a doubt. I have no interest in subsidizing lunches for those families that can afford to pay… I don’t see how it is fiscally responsible to provide every student with free meals regardless of family income level, just raise the minimum income level”. “This is the responsibility of parents/families, not a responsibility of our public schools”. “With the costs involved, I do not think the taxpayers need to feed those students whose families can afford it. I also think that the limits for qualifying should be raised at least $10 k. Instead of spending the money on feeding those that do not need it, the money should be allocated to teachers and facilities to improve the level of education”.

## Data Availability

The data presented in this study are available on request from the corresponding author. The data are not publicly available because the data are currently being utilized for additional analyses and ongoing research.

## Supplementary Materials

The following supporting information can be downloaded at: https://www.mdpi.com/article/10.3390/nu16020213/s1. File S1: School Community Perspectives Survey; File S2: Summary of closed-ended survey responses from all parents (*n* = 2347).
