# Supplementary material for: Parent Perceptions and Opinions of Universal Free School Meals in Arizona
Source: Nutrients. 2024 Jan 9;16(2):213. doi: 10.3390/nu16020213 (PMC10820707; doi:10.3390/nu16020213)
Supplement: Supplementary file 1 [file nutrients-16-00213-s001.zip › nutrients-2759411-supplementary.pdf]

**School Community Perspectives Survey**
Assessment of Healthy School Meals for All in Arizona
Conducted by ASU Food Policy and Environment Research Group, College of Health Solutions
Arizona State University

**Supplementary File S1: School Community Perspectives Survey**

**QConsent** You are invited to participate in a study sponsored by **XXX** to assess opinions about making school meals available to all students in Arizona at no cost. This short online survey will take approximately 15 minutes to complete. Your responses will help inform decision-makers as they plan for the future of school meal programs in **XXX**.

You must be 18 years or older to participate in the study and there are no foreseeable risks or discomforts to your participation. Your participation in this study is voluntary and you are free to withdraw from the study at any time. Your responses will be confidential. The results of this study may be used in reports, presentations, or publications, but your name will never be used.

At the end of the survey, you will have the option to provide your contact information if you would like to be entered into a drawing to win **X of Y $Z gift cards**.

If you have any questions concerning the research study, please contact the research team at **EMAIL** or **PHONE**. If you have any questions about your rights as a subject/participant in this research, or if you feel you have been placed at risk, you can contact the Chair of the Human Subjects Institutional Review Board, **IRB CONTACT INFORMATION**.

If you consent to take part in this study, please click “Yes, I consent” below. You will be automatically directed to the survey. If you do not wish to participate in the survey, simply close the survey page.

- Yes, I consent

**QRole_1 Are you an employee of a school or school district?**
Source: ASU Food Policy & Environment Research Group

1. Yes
2. No

**[Display if QRole_1 = Yes]**
**QRole_1.2 Which job title best describes your role in your school/district?**
Source: ASU Food Policy & Environment Research Group

1. Classroom teacher
2. Lunchroom staff/manager
3. School/district administrator
4. Other school/district support staff

**QRole1.3 Are you a parent of a child currently attending a K-12 school?**
Source: ASU Food Policy & Environment Research Group
1. Yes
2. No

***[Displayed to all participants]***

**Main_Text1**
**Background**
The National School Lunch Program provides nearly a million lunches and breakfasts for free or at a reduced-cost to school children in Arizona every day. These meals are financially supported with federal funding. To qualify for funding, school meals must meet nutrition standards, such as providing a variety of fruits and vegetables, and whole grains, limiting the use of salt, and following age-appropriate calorie limits. Prior to the onset of the COVID-19 pandemic, parents were required to submit income applications to determine their children's eligibility for free or reduced-cost meals. In response to the COVID-19 pandemic, schools were able to serve free school meals to all students nationwide using federal resources. This provision will no longer be in place as of the start of the 2022-2023 school year.
Source: KSHFNationalSurvey, modified

**Main_Text2:** In the following questions we would like your views about school meal programs. There are no right or wrong answers; we are interested in your honest opinion on these issues.

**Main_Fav Overall, how favorably do you view each of the following school meal programs?**
Source: HART, modified

Answer options: Very favorable, somewhat favorable, Neutral, Somewhat unfavorable, Very Unfavorable

1. The school lunch program
2. The school breakfast program

**Main_Gen How much do you agree with the following statements regarding school meals?**
Source: Cohen et al - Parent, modified

Answer options: Strongly disagree, Disagree, Neither agree nor disagree, Agree, Strongly agree

1. School meals save families money.
2. School meals reduce stress for families by saving time on preparing and packing meals.
3. Children are embarrassed to eat school meals.
4. School meals are only for children whose families have low incomes.
5. Eating school meals may benefit students academically.
6. School meals are healthy (i.e., meals are nutritious and balanced).

**Main_Spend How concerned are you about the amount the federal government spends on providing free or reduced-price school meals to students?**
Source: HART, modified

1. Extremely concerned
2. Moderately concerned
3. Somewhat concerned
4. Slightly concerned
5. Not at all concerned

**Main_ProFut Which of the following do you think would be the best approach to providing school meals in the future?**
Source: HART, modified

For reference, under the current income guidelines, a family of 3 earning more than $30,000 earns *too much* to qualify for free school meals

1. We should offer meals at no charge to all students who want them, regardless of income
2. We should raise the household income limits so more children qualify to receive free or reduced-cost meals
3. We should continue with the current income limits for free and reduced-cost meals
4. We should lower the household income limits so fewer children qualify to receive free or reduced-cost meals
5. No children should receive free or reduced-cost meals

**Main_SupFreeCovid How do you feel about the policy that was put into place during the COVID-19 pandemic that allowed public schools to serve school meals at no charge to all students?**
Source: HART, modified

1. Strongly support
2. Somewhat support
3. Neither support or oppose
4. Somewhat oppose
5. Strongly oppose

**Main_Text_3**

**Background**
To provide relief during the COVID-19 pandemic, federal legislation enabled schools to offer meals at no charge for all students. This policy ended for the current school year and families are again required to submit income applications. Only those children that meet the federal income guidelines will be eligible for free or reduced-cost school meals. The result is that some children, whose families make just over the income guidelines, lose access to these meals. Some states have passed legislation that keeps meals free for all students in their state.
Source:?

**Main_Benefits In your opinion, which of the following are the <u>most important benefits</u> of making school meals available at no charge to all students regardless of family income?**
Source: HART, modified

Choose up to three

1. Reduces child hunger
2. Removes major cost for low-income families
3. Reduces shame and stigma in the lunchroom
4. Improves academic achievement
5. Provides meals that are healthier than meals brought from home
6. Improves classroom behavior and school attendance
7. Decreases childhood obesity
8. Advances racial equity
9. Reduces paperwork burden for schools and families
10. Removes meal debt
11. Other, please specify

**Main_Sup Below is a list of potential reasons to <u>support</u> passing legislation to make school meals available to all students at no charge. How convincing are these reasons?**
Source: HART, modified

Answer options: Very convincing, somewhat convincing, Neither convincing or unconvincing, somewhat unconvincing, very unconvincing

1. A school meal at no charge often gives low-income children a nutritious meal that they might not otherwise get.
2. Many struggling families, including those with annual incomes as low as $30,000, don't currently qualify for free meals. This policy will help many families and children who are barely getting by.
3. School meals are important for academic success. Studies have shown participation in school meals improves students' attendance, behavior, and academic achievement.
4. School meals enhance child development and school readiness. Giving every child the option to get a healthy meal during the school day will help them be the best student they can be.
5. Offering school meals to all students at no charge will reduce the stigma associated with eating school meals and remove embarrassment due to unpaid school meal debt.

**Main_Opp Below is a list of potential reasons to <u>oppose</u> passing legislation to make school meals available to all students at no charge. How convincing are these reasons?**
Source: HART, modified

Answer options: Very convincing, somewhat convincing, Neither convincing or unconvincing, somewhat unconvincing, very unconvincing

1. The federal government loses billions of dollars due to improper payments and wasted food that is thrown away rather than eaten. We should fix these problems before expanding the school meal programs.
2. Students from wealthy backgrounds would be able to participate even though they can afford to pay for their lunch. Instead of helping well-off students, the program should be devoted to helping those in need.
3. The program will serve not only low-income children but all children whose parents won't prepare a home-packed meal for their child. It should be the responsibility of capable parents, not the school, to make sure their child is fed.
4. School meals at no charge would be an irresponsible use of taxpayer dollars. While schools around the country are already struggling to operate, we can't afford to give meals at no charge to every student.
5. Low-income children are already eligible for meals at no charge, but many don't participate. Instead of giving meals at no charge to students who can afford to buy lunch, the government should make school meals more nutritious and appealing.

**Main_SupOpp Review each statement below. In your opinion, is this a reason to support OR oppose making school meals available to all students at no charge in Arizona?**
Source: HART, modified

Answer options: Reason to support, not a reason either way, reason to oppose

1. Studies show that students who receive school meals eat more fruits, vegetables, and other healthy foods.
2. In Arizona, almost 65,000 children live in food insecure households and are not eligible for federal food assistance.

3. In schools that have made free school meals available to all students, the number of breakfasts served increased by approximately 10% and lunches by 5%.
4. Most students with family incomes above $30,000 do not currently qualify for free meals.
5. Before the COVID-19 pandemic, millions of students who qualified for free and reduced-cost meals did not participate because of stigma, administrative errors, and other barriers.

**Main_SupFreeAZ Would you <u>support or oppose</u> passing legislation in Arizona to permanently offer school meals at no charge to all students regardless of income?**
Source: HART, modified

1. Strongly support
2. Somewhat support
3. Neither support or oppose
4. Somewhat oppose
5. Strongly oppose

**PARENTS ONLY BLOCK**
**[Display if QRole1 = No and QRole1.3 = Yes]**

**Parents_SchType What type of school does your child attend?**

**If you have more than one child attending a K-12 school, please think of the child who had the most recent birthday when responding to these questions.**
Source: ASU Food Policy & Environment Research Group

1. Public school
2. Charter school
3. Private/Parochial school
4. Other ________________

**Parent_SchLevel Which of the following best describes the grades your child is in?**

*If you have more than one child attending a K-12 school, please think of the same child who had the most recent birthday when responding to this question.*
Source: HART, modified

1. Preschool/kindergarten
2. Elementary schools
3. Middle school or junior high
4. High School

**Parents_EatPre In a typical week <u>prior to the COVID-19 Pandemic, when schools were completely in-person (e.g., Fall of 2019)</u>, how often did your child eat school lunch (i.e., a lunch prepared in the school cafeteria and NOT a lunch brought from home)?**
Source: Cohen et al - Parent, modified

*If you have more than one child attending school, please think of the same child who had the most recent birthday who is in grade K-12 when responding to this question.*
1. Never
2. One day per week
3. Two days per week
4. Three days per week

5. Four days per week
6. Five days per week
7. Not sure
8. My child was not school-age during the 2019 - 2020 school year

**Parents_EatPost In a typical week, <u>during the previous school year (2021-2022) when school meals were offered at no charge to all students</u>, how often did your child eat school lunch (i.e., a lunch prepared in the school cafeteria and NOT a lunch brought from home)?**
Source: HART, modified

If you have more than one child attending school, please think of the same child who had the most recent birthday who is in grade K-12 when responding to this question.

1. Never
2. One day per week
3. Two days per week
4. Three days per week
5. Four days per week
6. Five days per week
7. Not sure
8. My child was not school-age during the 2021 - 2022 school year

***[Display if Never is NOT selected in*** **Parents_EatPre OR Never is NOT selected in Parents_EatPost]**

**Parents_PartY What are the main reasons that your child eats school meals?**
Source: ASU Food Policy & Environment Research Group

Please select all that apply.

1. Most children in my child's school eat school meals.
2. My child likes eating school meals.
3. School meals help my family save money.
4. School meals help me and my family save time.
5. School meals are healthy.
6. School meals provide enough food for my child to feel full.
7. Other, please specify

***[Display if Five days per week is NOT selected in*** **Parents_EatPre OR** ***Five days per week*** **is NOT selected in Parents_EatPost]**

**Parents_PartN What are the main reasons that your child may *<u>not</u>* eat school meals?**
Source: ASU Food Policy & Environment Research Group/used answer options for Cohen et al - Parents

Please select all that apply.

1. The meal application process is time-consuming and/or confusing.
2. My child does not like the taste of school meals.
3. My child gets tired of the same foods being served in school meals.
4. I have concerns about the healthfulness of school meals.
5. Prior to the COVID-19 pandemic, the cost of meals was too high.
6. My child does not have time to eat school meals.
7. My child does not get enough food in school meals to feel full.

8. My child is embarrassed to eat school meals.
9. School meals are for low-income children only.
10. Other, please specify

**FOODSERVICE BLOCK**

***[Display if* QRole_1= YES and QRole_1.2 = 2*]***

**FS_SchType What type of school do you work for?**
Source: ASU Food Policy & Environment Research Group

1. Public school
2. Charter school
3. Private/Parochial school
4. Other ________________

**FS_Impact In your opinion, what was the impact on your school meal program as a result of providing school meals to all students at no charge during the 2021-22 school year?**
Source: Cohen, et al & Zuercher, et al – FSD

Answer options: Decreased greatly, decreased slightly, no effect, increased slightly, increased greatly

1. Student meal participation
2. Paperwork/administrative burden (e.g., collecting Meal Application forms, tracking student eligibility in line, etc.)
3. Time in line for students to get meals
4. Crowding in student dining areas
5. Stigma for students eating school meals
6. Unpaid meal charges/debt
7. School food waste
8. School meal packaging/solid waste
9. Foodservice staffing challenges
10. Scratch/modified scratch cooking
11. Parent satisfaction with meals offered

**FS_Con1 If Arizona were to make school meals available to all students at no charge in a future school year, how concerning, if at all, are the following?**
Source: Cohen, et al & Zuercher, et al – FSD

Answer options: Significant concern, Moderate concern, Minimal concern, Not a concern

1. Loss of sales from a la carte
2. Inadequate product or ingredient availability
3. Difficulties meeting school meal nutrition standards
4. Difficulties maintaining meal quality and variety
5. Staffing shortages
6. Lack of time for staff training
7. Inadequate kitchen equipment
8. Inadequate kitchen facility and/or storage space
9. Increases in school meal food waste
10. Increases in school meal packaging/solid waste
11. Increased time in line for students to get meals
12. Inadequate meal service space
13. Inadequate dining space

14. Not enough time for students to eat

**FS_Con2 If Arizona were to make school meals available to all students at no charge in a future school year, how concerning, if at all, are the following?**
Source: Cohen, et al & Zuercher, et al – FSD

Answer options: Significant concern, Moderate concern, Minimal concern, Not a concern

1. Increase in student and parent complaints
2. Difficulties in meeting student cultural/ethnic food preferences
3. Difficulties in meeting student food allergies/medical nutrition needs
4. Difficulty obtaining income information from families
5. Lack of support from district administration (school board, superintendent)
6. Lack of support from school administration (principals/vice principals)
7. Lack of support from nutrition services staff
8. Lack of support from classroom teachers, school nurses, and other school personnel
9. Lack of support from school or district wellness committees

**FS_Part Thinking about the students who did <u>NOT</u> regularly eat the reimbursable school meals last year (SY 2021-22), how common are the following barriers for the students you serve?**
Source: Cohen, et al & Zuercher, et al – FSD, modified

Answer options: Not at all common, slightly common, somewhat common, moderately common, extremely common

1. Students do not like the taste of the food
2. Students or parents do not think the food is healthy
3. Students prefer to eat a la carte options
4. Students get tired of the foods served at lunch
5. Meals do not meet students' cultural preferences
6. Portions are not big enough / not enough food provided
7. Students prefer to eat meals from home or elsewhere
8. Students often skip meals (e.g., do not eat any breakfast or lunch)
9. Students are unable to get to school on time for breakfast
10. Students don't have enough time to get and eat the lunch
11. Students or parents think only low-income kids eat school meals
12. Students' friends don't eat the school meals
13. Other, please specify

**FS_Benefits In your opinion, which of the following would be the <u>most important benefits to your food service program</u> if school meals were available at no charge to all students regardless of family income?**
Source: ASU Food Policy & Environment Research Group

Choose up to two

1. Increased revenue due to more student participation
2. Reduced time spent tracking student eligibility status at mealtimes
3. Reduced time spent tracking and collecting meal debt
4. Faster meal service
5. More time to focus on meal quality
6. Other, please specify

**TEACHER BLOCK**

***Display if* QRole_1= YES and QRole_1.2 = 1]**

**Teach_SchType What type of school do you work for?**
Source: ASU Food Policy & Environment Research Group

1. Public school
2. Charter school
3. Private/Parochial school
4. Other ________________

**Teach_1 Over the past two school years, when school meals were available to all students at no cost regardless of family income, did you see more, less or about the same of the following?**
Source: ASU Food Policy & Environment Research Group

Answer options: more, less, about the same, this has never been an issue in my classroom

1. Disruptive classroom behavior
2. Hungry students in the classroom
3. Children seeking food from alternative sources (such as out of the trash, seeking food from classmates or teachers, etc)

**Teach_2 If school meals were available at no charge to all students in your school regardless of family income, how concerning, if at all, are the following issues?**
Source: ASU Food Policy & Environment Research Group

Answer options: Significant concern, Moderate concern, Minimal concern, Not a concern

1. Children will eat more than what they need
2. The program will negatively impact overall school funding
3. There will not be enough time for students to get lunch
4. The timing/length of the school day will change
5. There will be an increase in food waste

**ADMINISTRATOR OR OTHER SUPPORT STAFF BLOCK**

**Admin_SchType What type of school do you work for?**
Source: ASU Food Policy & Environment Research Group

1. Public school
2. Charter school
3. Private/Parochial school
4. Other ________________

**Admin_Concern If school meals were available at no charge to all students in your school or district regardless of family income, how concerning, if at all, are the following issues?**
Source: ASU Food Policy & Environment Research Group

Answer options: Significant concern, Moderate concern, Minimal concern, Not a concern

1. Children will eat more than what they need
2. The program will negatively impact overall school funding
3. There will not be enough time for students to get lunch
4. The timing/length of the school day will change
5. There will be an increase in food waste

***[Displayed to all participants]***

**Open Please provide any additional thoughts, opinions, or experiences you may have about offering school meals at no charge to all Arizona students regardless of family income.**

| |
|---|
| |

**Demo_Text_1: Now we are going to ask you a few questions for statistical purposes only.**
Source: KSHFNationalSurvey

**Demo_EDU What is the highest level of education you have completed?**
Source: Cohen - Parents

1. Less than high school
2. High school graduate
3. Some college
4. 2-year degree
5. 4-year degree
6. Professional degree
7. Doctorate

**Demo_Political In terms of your views on political issues, how would you describe yourself?**

1. Very conservative
2. Somewhat conservative
3. Middle of the road
4. Somewhat liberal
5. Very liberal
6. Not sure

**Demo_ZipCode What is your zip code?**

**Demo_Age What age group do you belong to?**
1. 18-34
2. 35-54
3. 55 or older

**Demo_Eth What is your Ethnicity:**
1. Hispanic/Latino
2. NOT Hispanic/Latino

**Demo_Race What is your race (check all that apply):**
1. White
2. Black/African American
3. American Indian/Alaska Native
4. Asian/Asian American
5. Native Hawaiian/ Pacific Islander
6. Other (please specify)

**Demo_HHA How many people are in your household?**

1 2 3 4 5 6 7 8 9+

**Demo_HHC How many total children under 18 years old live in your household?**

0 1 2 3 4 5 6 7 8 9+

**Demo_Income What was your household income before taxes in the past 12 months? It is ok to make your best guess.**

1. Less than $19,999
2. $20,000 - $24,999
3. $25,000 - $34,999
4. $35,000 - $49,999
5. $50,000 - $64,999
6. $65,000 - $79,999
7. $80,000 - $99,999
8. $100,000 - $149,999
9. $150,000 or more

**Drawing_YN** Thank you for taking the time to complete this survey.

Would like to be entered into the drawing for a chance to win a $100 gift card?

- Yes
- No

**[Display if Drawing_YN Yes is selected]**

**Drawing_EM** Please enter your email address to be entered into the drawing for the gift card. We will only use this email address to contact you if you are selected as a winner of the gift card.

Email:

**Interview_YN** Would it be ok to contact you for a brief (online) interview after you complete this survey so that we can learn more about your opinions of school meals? In addition to having the chance to win a $100 gift card for the current survey, <u>you will automatically receive a $50 gift card</u> for your time (and interviews can be scheduled at a day and time that you choose).

1. Yes
2. No

**[Display if Interview_YN YES is selected AND if Drawing_YN NO is selected]**

**Interview_EM** Please provide your email address. We will only use this email address to contact you to schedule an interview and to email the gift card as a thank you for your participation in the interview

Email:

**[Display if Interview_YN YES is selected]**

**Interview_Phone** Please provide your phone number.

We will use this as a backup to contact you about a) scheduling an interview, and/or winning the gift card for your survey participation and/or for brief reminders before your scheduled interview. Your phone number will not be shared with anyone and will only be used for the reasons noted above. You can opt out of SMS text reminders at any time.

If you do not want to receive SMS text reminders, you may leave this space blank.

Phone number:

| Source Citations | |
|---|---|
| HART | Molyneux, Guy. Building Momentum for Healthy School Meals for All. Panel presentation at: National Anti-Hunger Policy Conference: March, 2022, Virtual.<br><br>**Guy Molyneux co-presented with Dr. Janet Poppendieck – CUNY Urban Food Policy Institute and Maria Martiosayn – Office of Rep. Ilhan Omar<br><br>Questions were taken from the slides Guy presented during this session |
| Cohen, et al – Parents | Parent survey provided by Healthy School Meals for All work groups. The survey was used to survey parents in Ca and Me with the goal to assess parent perceptions of UFSM and barriers/motivators to completing school meal application forms<br><br>The original survey can be found at: https://www.childnourishlab.org/healthy-school-meals-for-all |
| Cohen, et al & Zuercher, et al – FSD | Food service directors survey provided by the Healthy School Meals for All workgroup. The survey was used to document the district foodservice director's perspectives about the opportunities and challenges presented by FSMFA to inform initial implementation<br><br>Cohen, J.F., Polacsek, M., Hecht, C.E., Hecht, K., Read, M., Olarte, D.A., Patel, A.I., Schwartz, M.B., Turner, L., Zuercher, M. and Gosliner, W., 2022. Implementation of Universal School Meals during COVID-19 and beyond: Challenges and Benefits for School Meals Programs in Maine. Nutrients, 14(19), p.4031.<br><br>Zuercher, M.D., Cohen, J.F., Hecht, C.E., Hecht, K., Ritchie, L.D. and Gosliner, W., 2022. Providing school meals to all students free of charge during the COVID-19 pandemic and beyond: Challenges and benefits reported by school foodservice professionals in California. Nutrients, 14(18), p.3855.<br><br>The original survey can be found at: https://www.childnourishlab.org/healthy-school-meals-for-all |
| ASU Food Policy & Environment Research Group | Original questions developed by researchers in the ASU Food Policy & Environment Research Group at ASU's College of Health Solutions |
| KSHFNationalSurvey | Kids' Safe and Healthful Food Project. The Kids' Safe and Healthful Foods Project provides nonpartisan analysis and evidence-based recommendations on policies that affect the safety and healthfulness of school foods. The project is a collaboration between The Pew Charitable Trusts and the Robert Wood Johnson Foundation.<br><br>Original survey/survey results can be found at: https://www.pewtrusts.org/-/media/assets/2014/09/kshfnationalsurvey_raw.pdf |

**Supplementary File S2: Summary of closed-ended survey responses from all parents (*n*= 2347)**

**Figure S2.1: Parents' view of school breakfast and school lunch**

Question text: Overall, how favorably do you view each of the following school meal programs?

Answer options: Very favorable, somewhat favorable, neutral, somewhat unfavorable, very unfavorable. Very favorable and somewhat favorable were combined into "favorable" while somewhat unfavorable and very unfavorable were combined into "unfavorable".

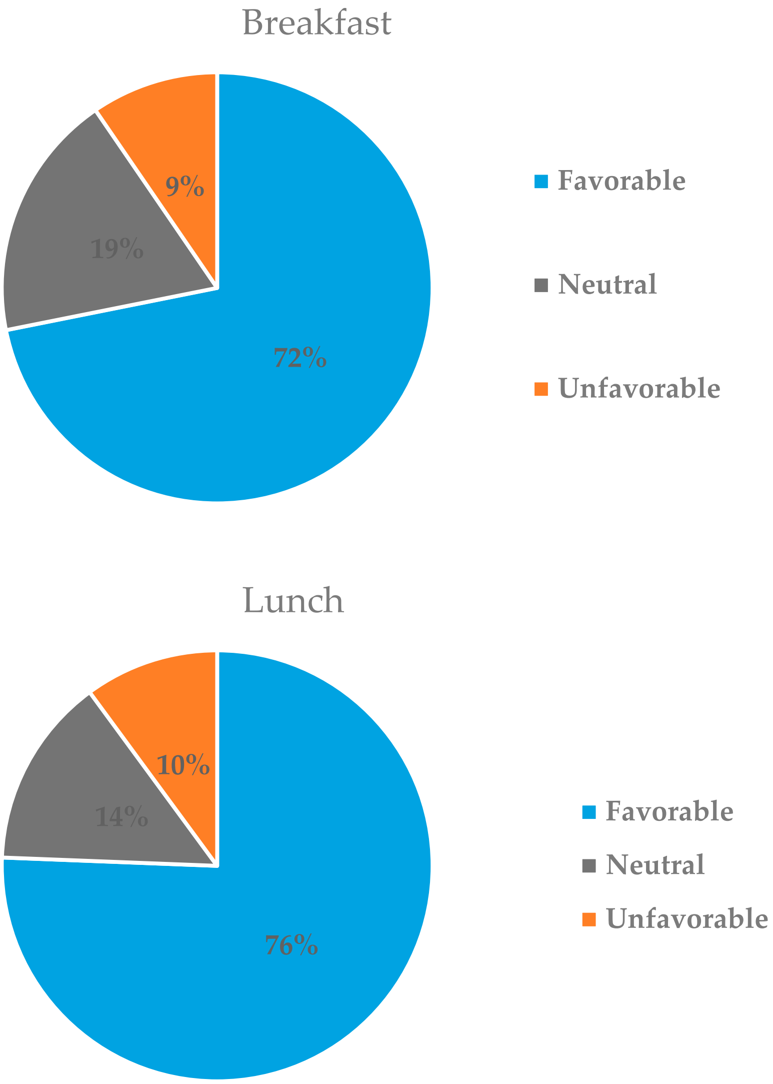

**Table S2.1: Percent and total number of parents that disagreed, neither agreed nor disagreed, or agreed with each statement about school meals**

Question text: How much do you agree with the following statements regarding school meals?

Answer options: Strongly disagree, disagree, neither agree nor disagree, agree, strongly agree. Strongly disagree and disagree were combined into "disagree" and agree and strongly agree were combined into "agree".

| | Disagree N % | Neither N % | Agree N % |
|---|---|---|---|
| School meals save families money | 342 (14.6) | 185 (7.5) | 1818 (77.5) |
| l meals reduce stress for families by saving time on preparing and packing meals | 255 (10.9) | 113 (4.8) | 1971 (84.3) |
| Children are embarrassed to eat school meals | 1294 (55.4) | 643 (27.5) | 332 (14.2) |
| School meals are only for children whose families have low incomes | 1695 (74.4) | 332 (14.2) | 313 (13.4) |
| Eating school meals may benefit students academically | 209 (8.9) | 391 (16.7) | 1741 (74.4) |
| School meals are healthy (i.e., meals are nutritious and balanced) | 581 (24.8) | 437 (18.7) | 1321 (56.5) |

**Figure S2.2: Parents' reported level of concern about government spending on school meals**

Question text: How concerned are you about the amount the federal government spends on providing free or reduced-price school meals to students?

Answer options: Extremely concerned, moderately concerned, somewhat concerned, slightly concerned, not at all concerned.

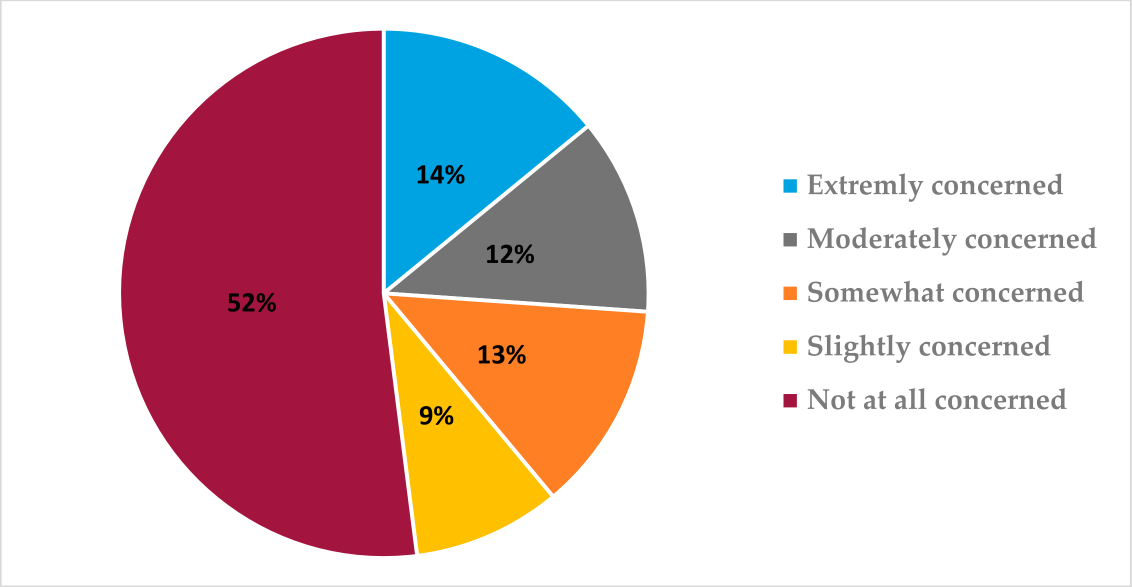


**Table S2.2: Parents' opinions about the best approach to provide school meals in the future**

Question text: Which of the following do you think would be the best approach to providing school meals in the future?
For reference, under the current income guidelines, a family of 3 earning more than $30,000 earns too much to qualify for free school meals.

| | N % |
|---|---|
| We should offer meals at no charge to all students who want them, regardless of income | 1913 (81.5) |
| We should raise the household income limits so more children qualify to receive free or reduced-cost n | 334 (14.2) |
| We should continue with the current income limits for free and reduced-cost meals | 79 (3.4) |
| We should lower the household income limits so fewer children qualify to receive free or reduced-cost | 9 (0.4) |
| No children should receive free or reduced-cost meals | 8 (0.3) |

**Figure S2.3: Parents' reported support for the policy that was put in place during the COVID-19 pandemic that allowed public schools to serve meals at no charge to all students**

Question text: How do you feel about the policy that was put into place during the COVID-19 pandemic that allowed public schools to serve school meals at no charge to all students?

Answer options: Strongly support, somewhat support, neither support or oppose, somewhat oppose, strongly oppose. Strongly support and somewhat support were combined into "support" and somewhat oppose and strongly oppose were combined into "oppose".

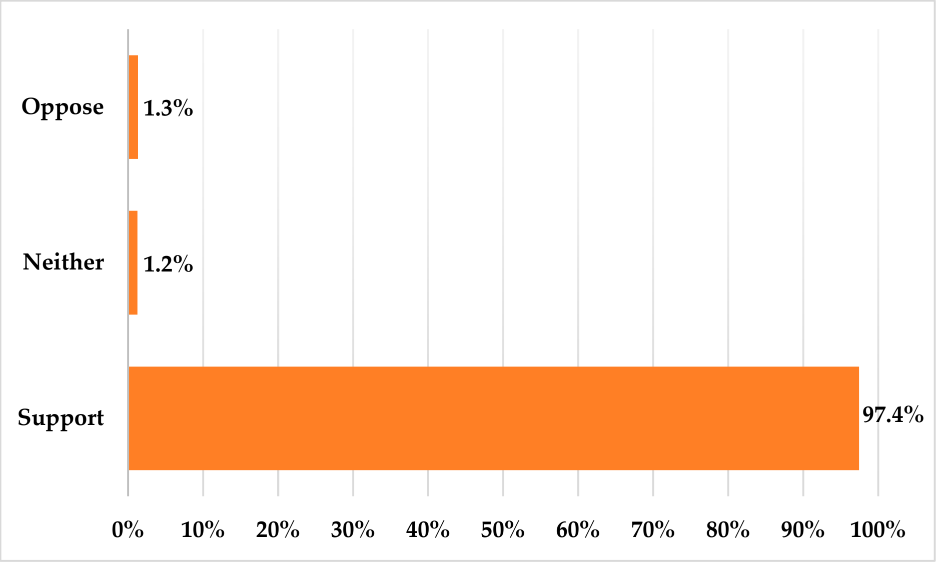


**Table S2.3: Summary of parents' views on how convincing or unconvincing specific messages are for supporting HSM4A legislation**

Question text: Below is a list of potential reasons to support passing legislation to make school meals available to all students at no charge. How convincing are these reasons?

Answer options: Very convincing, somewhat convincing, neither convincing nor unconvincing, somewhat unconvincing, very unconvincing. Very convincing and somewhat convincing were combined into "convincing" and unconvincing and somewhat unconvincing were combined into "unconvincing".

| | Convincing | | Neither Convincing or Unconvincing | | Unconvincing | |
|---|---|---|---|---|---|---|
| | N | (%) | N | (%) | N | (%) |
| A school meal at no charge often gives low-income children a nutritious meal that they might not otherwise get. | 2179 | (93.5) | 86 | (3.7) | 66 | (2.8) |
| Many struggling families, including those with annual incomes as low as $30,000, don't currently qualify for free meals. This policy will help many families and children who are barely getting by. | 2131 | (91.2) | 120 | (5.1) | 86 | (3.7) |
| School meals are important for academic success. Studies have shown participation in school meals improves students' attendance, behavior, and academic achievement. | 2140 | (91.6) | 130 | (5.6) | 66 | (2.8) |
| School meals enhance child development and school readiness. Giving every child the option to get a healthy meal during the school day will help them be the best student they can be. | 2160 | (92.6) | 107 | (4.6) | 66 | (2.8) |
| Offering school meals to all students at no charge will reduce the stigma associated with eating school meals and remove embarrassment due to unpaid school meal debt. | 2057 | (88.1) | 173 | (7.4) | 106 | (4.5) |

**Table S2.4: Summary of parents' views on how convincing or unconvincing specific messages are for opposing HSM4A legislation**

Answer options: Below is a list of potential reasons to <u>oppose</u> passing legislation to make school meals available to all students at no charge. How convincing are these reasons?

Answer options: Very convincing, somewhat convincing, Neither convincing nor unconvincing, somewhat unconvincing, very unconvincing. Very convincing and somewhat convincing were combined into "convincing" and unconvincing and somewhat unconvincing were combined into "unconvincing".

| | Convincing | | Neither Convincing or Unconvincing | | Unconvincing | |
|---|---|---|---|---|---|---|
| | N | (%) | N | (%) | N | (%) |
| The federal government loses billions of dollars due to improper payments and wasted food that is thrown away rather than eaten. We should fix these problems before expanding the school meal programs. | 993 | (42.5) | 387 | (16.6) | 955 | (40.9) |
| Students from wealthy backgrounds would be able to participate even though they can afford to pay for their lunch. Instead of helping well-off students, the program should be devoted to helping those in need. | 829 | (35.5) | 534 | (22.9) | 970 | (41.6) |
| The program will serve not only low-income children but all children whose parents won't prepare a home-packed meal for their child. It should be the responsibility of capable parents, not the school, to make sure their child is fed. | 613 | (26.3) | 368 | (15.8) | 1353 | (58.0) |
| School meals at no charge would be an irresponsible use of taxpayer dollars. While schools around the country are already struggling to operate, we can't afford to give meals at no charge to every student. | 436 | (18.7) | 301 | (12.9) | 1596 | (68.4) |
| Low-income children are already eligible for meals at no charge, but many don't participate. Instead of giving meals at no charge to students who can afford to buy lunch, the government should make school meals more nutritious and appealing. | 1111 | (47.6) | 451 | (19.3) | 771 | (33.1) |

**Table S2.5: Summary of parents' views on if specific messages are a reason to support or oppose HSM4A legislation**

Question text: Review each statement below. In your opinion, is this a reason to support OR oppose making school meals available to all students at no charge in Arizona?

Answer options: Reason to support, not a reason either way, reason to oppose

| | Reason to Support | | Not a reason either way | | Reason to oppose | |
|---|---|---|---|---|---|---|
| | N | (%) | N | (%) | N | (%) |
| Studies show that students who receive school meals eat more fruits, vegetables, and other healthy foods. | 2024 | (86.7) | 281 | (12.0) | 29 | (1.2) |
| In Arizona, almost 65,000 children live in food insecure households and are not eligible for federal food assistance. | 2173 | (93.1) | 139 | (6.0) | 23 | (1.0) |
| In schools that have made free school meals available to all students, the number of breakfasts served increased by approximately 10% and lunches by 5%. | 1924 | (82.5) | 376 | (16.1) | 31 | (1.3) |
| Most students with family incomes above $30,000 do not currently qualify for free meals. | 1708 | (73.5) | 471 | (20.3) | 145 | (6.2) |
| Before the COVID-19 pandemic, millions of students who qualified for free and reduced-cost meals did not participate because of stigma, administrative errors, and other barriers. | 1593 | (68.8) | 633 | (27.3) | 91 | (3.9) |

**Figure S2.4: Parents' reported support for offering HSM4A in Arizona**

Question text: Would you support or oppose passing legislation in Arizona to permanently offer school meals at no charge to all students regardless of income?

Answer options: Strongly support, somewhat support, neither support or oppose, somewhat oppose, strongly oppose. Strongly support and somewhat support were combined into "support" and Somewhat oppose and strongly oppose were combined into "oppose".

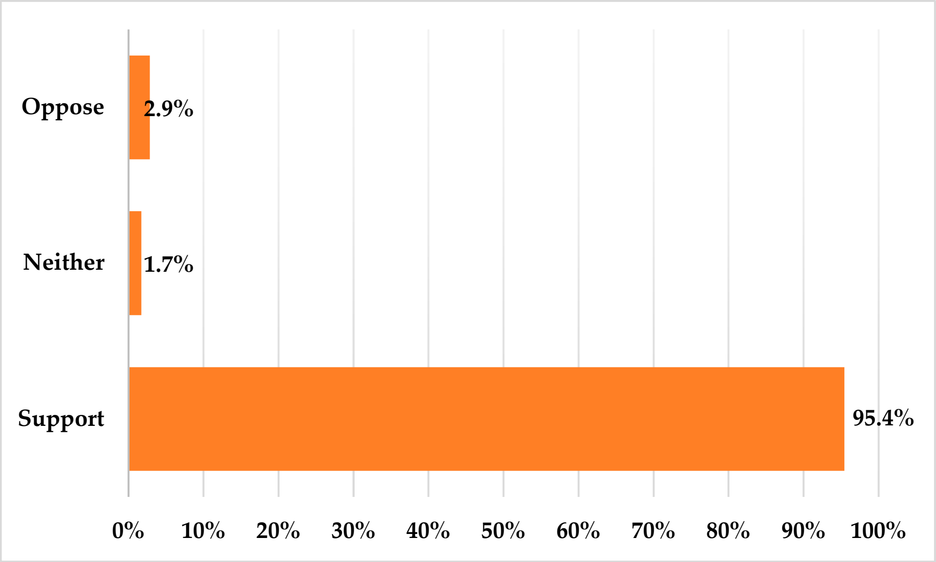


**Table S2.6: Summary of the types of schools parents reported their students attending**

Question text: What type of school does your child attend? If you have more than one child attending a K-12 school, please think of the child who had the most recent birthday when responding to these questions.

Answer options: Public school, Charter school, Private/Parochial school, Other

| | N | % |
|---|---|---|
| School Type | | |
| Public | 2284 | (91.3) |
| Charter | 27 | (1.2) |
| Private | 13 | (0.6) |
| Other | 17 | (0.7) |

**Table S2.7: Summary of parents' report of grades children attended at the time of the survey**

Question text: Which of the following best describes the grades your child is in?

Answer options: Preschool/Kindergarten, Elementary school, Middle school or junior high, High school

| | N | % |
|---|---|---|
| School Level | | |
| Pre K/Kindergarten | 203 | (8.7) |
| Elementary | 1070 | (45.6) |
| Middle | 438 | (18.7) |
| High School | 632 | (29.9) |

**Figure S2.5: Parents' reported participation of their child in school meals prior to the Covid-19 pandemic**

Question text: In a typical week <u>prior to the COVID-19 Pandemic, when schools were completely in-person (e.g., Fall of 2019)</u>, how often did your child eat school lunch (i.e., a lunch prepared in the school cafeteria and NOT a lunch brought from home)?

Answer options: Never, one day per week, two days per week, 3 days per week, 4 days per week, 5 days per week, not sure, my child was not school-age during the 2019-2020 school year.

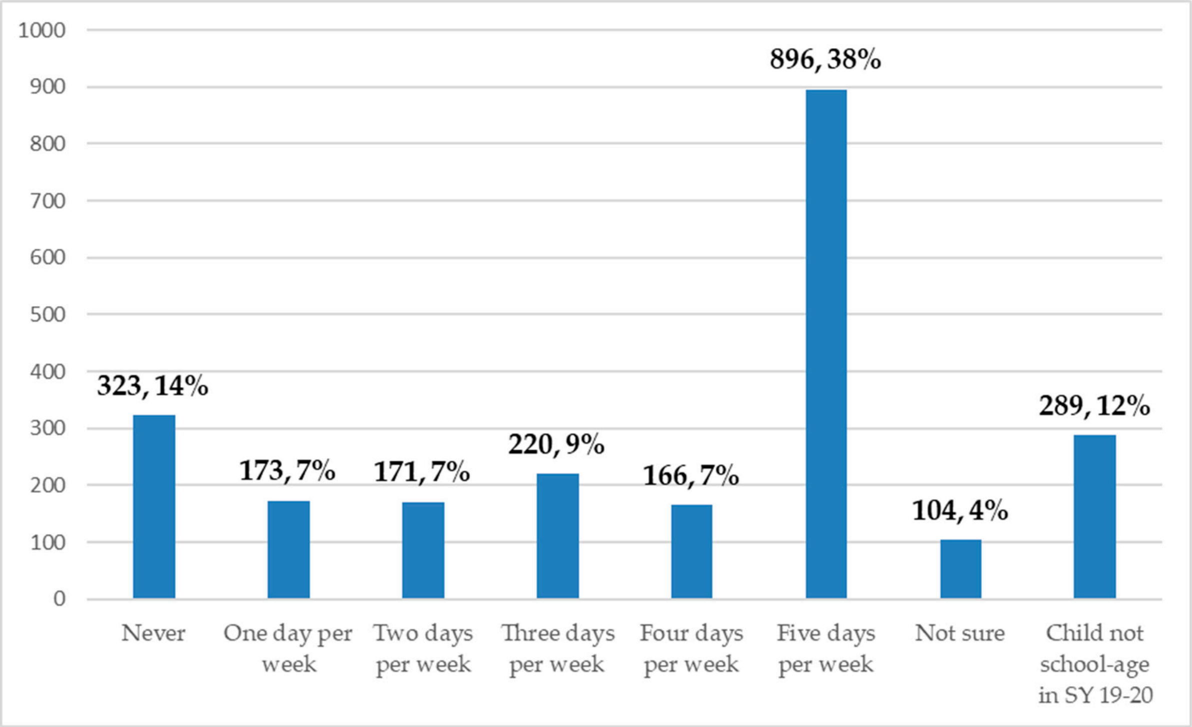

**Figure S2.6: Parents' reported participation of their child in school meals when Covid-19 pandemic waivers were in place (school year 2021-2022)**

Question text: In a typical week, during the previous school year (2021-2022) when school meals were offered at no charge to all students, how often did your child eat school lunch (i.e., a lunch prepared in the school cafeteria and NOT a lunch brought from home)? If you have more than one child attending school, please think of the same child who had the most recent birthday who is in grade K-12 when responding to this question.

Answer options: Never, one day per week, two days per week, 3 days per week, 4 days per week, 5 days per week, not sure, my child was not school-age during the 2021-2022 school year.

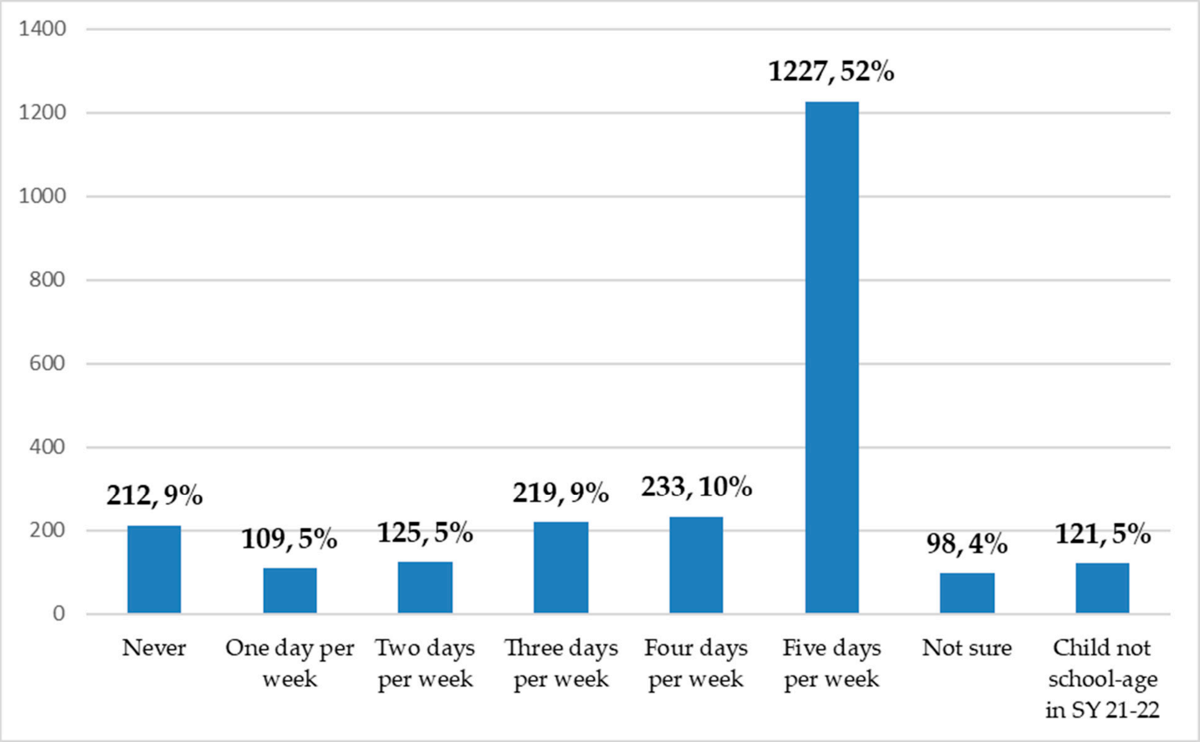

**Table S2.8: Parents' reported reasons for participating in school meals**

Question text: What are the main reasons that your child eats school meals? Please select all that apply.

| | N | (%) |
|---|---|---|
| School meals help me and my family save time | 1294 | (58.7) |
| School meals help my family save money | 1279 | (58.1) |
| My child likes eating school meals | 1144 | (51.9) |
| School meals provide enough food for my child to feel full | 762 | (34.6) |
| Most children in my child's school eat school meals | 754 | (34.2) |
| School meals are healthy | 673 | (30.6) |

**Table S2.9: Parents' reported reasons for not participating in school meals**

Question text: What are the main reasons that your child may not eat school meals? Please select all that apply.

| | N | (%) |
|---|---|---|
| My child does not like the taste of school meals | 718 | (46.4) |
| My child gets tired of the same foods being served in school meals | 430 | (27.8) |
| I have concerns about the healthfulness of school meals | 327 | (21.1) |
| Prior to the COVID-19 pandemic, the cost of meals was too high | 276 | (17.8) |
| My child does not have time to eat school meals | 234 | (15.1) |
| The meal application process is time-consuming and/or confusing | 207 | (13.4) |
| My child does not get enough food in school meals to feel full | 175 | (11.3) |
| My child is embarrassed to eat school meals | 151 | (9.8) |
| School meals are for low-income children only | 144 | (9.3) |
